# The dark art of light measurement: accurate radiometry for low-level light therapy

**DOI:** 10.1007/s10103-016-1914-y

**Published:** 2016-03-10

**Authors:** Mohammed A. Hadis, Siti A. Zainal, Michelle J. Holder, James D. Carroll, Paul R. Cooper, Michael R. Milward, William M. Palin

**Affiliations:** Biomaterials Unit, School of Dentistry, College of Medical and Dental Sciences, University of Birmingham, St Chads Queensway, Birmingham, UK B4 6NN; Oral Biology, School of Dentistry, College of Medical and Dental Sciences, University of Birmingham, St Chads Queensway, Birmingham, UK B4 6NN; THOR Photomedicine Ltd, Chesham, UK

**Keywords:** Radiometry, Low-level light therapy, Low-level laser therapy, LLLT, Photobiomodulation

## Abstract

Lasers and light-emitting diodes are used for a range of biomedical applications with many studies reporting their beneficial effects. However, three main concerns exist regarding much of the low-level light therapy (LLLT) or photobiomodulation literature; (1) incomplete, inaccurate and unverified irradiation parameters, (2) miscalculation of ‘dose,’ and (3) the misuse of appropriate light property terminology. The aim of this systematic review was to assess where, and to what extent, these inadequacies exist and to provide an overview of ‘best practice’ in light measurement methods and importance of correct light measurement. A review of recent relevant literature was performed in PubMed using the terms LLLT and photobiomodulation (March 2014–March 2015) to investigate the contemporary information available in LLLT and photobiomodulation literature in terms of reporting light properties and irradiation parameters. A total of 74 articles formed the basis of this systematic review. Although most articles reported beneficial effects following LLLT, the majority contained no information in terms of how light was measured (73 %) and relied on manufacturer-stated values. For all papers reviewed, missing information for specific light parameters included wavelength (3 %), light source type (8 %), power (41 %), pulse frequency (52 %), beam area (40 %), irradiance (43 %), exposure time (16 %), radiant energy (74 %) and fluence (16 %). Frequent use of incorrect terminology was also observed within the reviewed literature. A poor understanding of photophysics is evident as a significant number of papers neglected to report or misreported important radiometric data. These errors affect repeatability and reliability of studies shared between scientists, manufacturers and clinicians and could degrade efficacy of patient treatments. Researchers need a physicist or appropriately skilled engineer on the team, and manuscript reviewers should reject papers that do not report beam measurement methods and all ten key parameters: wavelength, power, irradiation time, beam area (at the skin or culture surface; this is not necessarily the same size as the aperture), radiant energy, radiant exposure, pulse parameters, number of treatments, interval between treatments and anatomical location. Inclusion of these parameters will improve the information available to compare and contrast study outcomes and improve repeatability, reliability of studies.

## Introduction

‘Low-level light therapy’ (LLLT) or the recently accepted Medical Subject Heading (MeSH) term, photobiomodulation is the application of light typically within the wavelength range ~600–1000 nm to directly stimulate or inhibit cellular and biological processes. The application of low power (<500 mW; non-thermal and non-destructive) lasers or light-emitting diodes (LEDs; or even a combination of both) have shown therapeutic effects with a number of light parameters that include irradiance, exposure time and total energy delivered.

Many studies have reported beneficial effects of LLLT following trauma in improving tissue healing [1], reducing inflammation [2], reducing oedema [3], restoring blood flow [4] and inducing analgesia [5] in a number of medical specialties that include musculoskeletal injuries, skin diseases, degenerative diseases, neuropathic pain syndromes and even traumatic brain injuries [1–8]. Favourable data for LLLT in other biomedical areas now also exists, which includes several dental specialties such as endodontics, maxillofacial surgery, oral pathology, oral surgery, orthodontics, pediatric, periodontics and prosthodontics [9] for a range of conditions including oral mucositis [10], dentine hypersensitivity [11] and candidiasis [12]. The application of LLLT may also prevent pain and protect muscles prior to strenuous exercise or trauma, which has significant implications for the wider use of this therapeutic technology as a pre-conditioning modality prior to surgical procedures [13].

Despite several thousand *in vitro* studies, *in vivo* studies and clinical trials reporting positive beneficial effects, articles exist where nil or negative effects have been reported, promoting controversy surrounding the effectiveness of LLLT [14–18]. In certain studies, non-significant effects can be attributed to several factors relating to dosimetry; too much or too little energy, irradiance and exposure time as well as pulse structure and insufficient irradiation area [14, 19, 20]. It is clear that there is a therapeutic window in terms of dosimetry and a biphasic dose response which has been likened to the Arndt-Schulz or hormesis curve [19]. Consequently, irradiation parameters are likely to be key to whether outcomes have a positive, nil or negative effect. Although LLLT parameters are known and have been previously defined in the literature, including specialised mandatory and volunteer laser safety international standards such as US Code of Federal Regulations, American National Standards Institute and the International Standards Manual and other laser safety books and review articles [21, 22], beam parameters are often not measured, calibration of measuring instruments are rarely verified, critical data is often unreported, and in some cases, there are elementary dose calculation errors, all of which leading to misinformation in the literature. The importance of correct measurement and reporting has been emphasised several times within the literature [23–28] and brief ‘guidelines’ on how to measure and report LLLT dose and beam parameters in clinical and laboratory studies has also been published [23, 29, 30].

The aims of this work are to (1) review the adequacy of reporting irradiation parameters in recent literature, (2) describe fundamental concepts and appropriate methodology for best practice in light property evaluation and (3) define the correct terminology for reporting radiometric parameters.

## Methods

**Fig. 1:**
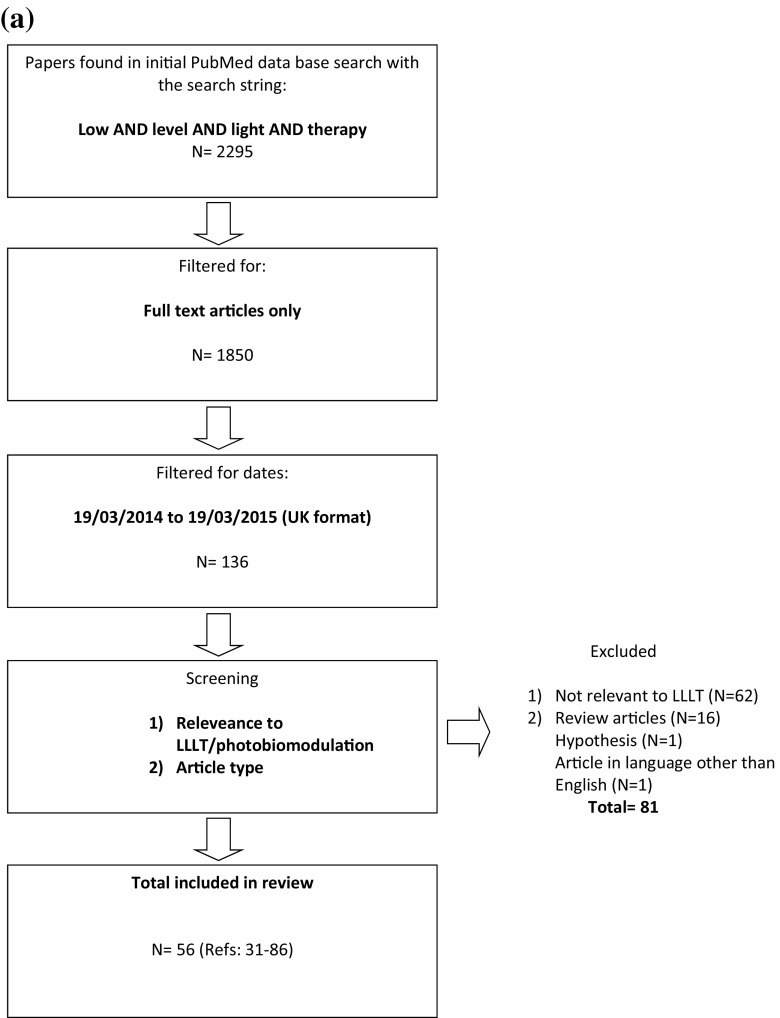

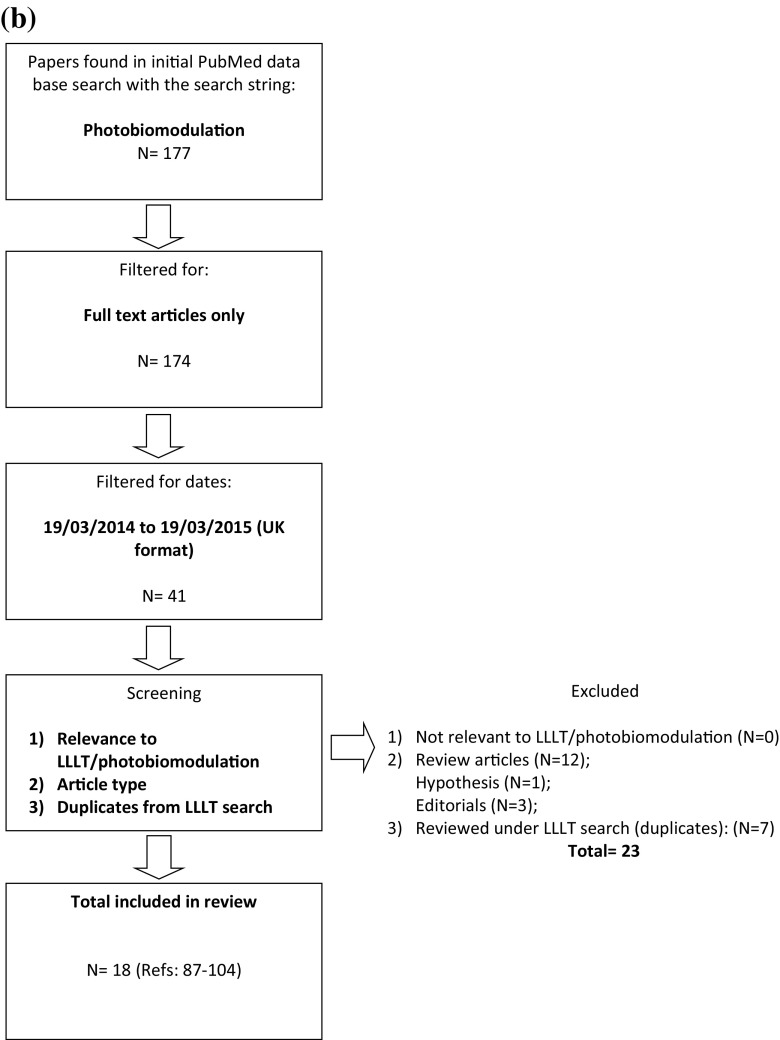
**a** Flow chart of search strategy to identify articles for review using ‘low and level and light and therapy.’ **b** Flow chart of search strategy to identify articles for review using ‘photobiomodulation’

To assess the methods and variability of measuring and reporting LLLT irradiation parameters, and radiometric terminology used by researchers, a review of recent relevant literature was performed in PubMed. The following two searches where performed separately: (low and level and light and therapy) and (photobiomodulation). These specific search terms were used as ‘LLLT’ has been widely recognised and used as a MeSH term for many years, although more recently photobiomodulation has become accepted as a more appropriate description of the action of light on cellular behaviour. Following the literature searches, the results were filtered for ‘full-text article’, published in a 1-year period between 19th March 2014 and 19th March 2015 (Fig. 1) to ensure a manageable number of articles whilst assessing the latest methods used by researchers. Review articles, editorials, articles in languages other than English and those not relevant to LLLT or photobiomodulation, were excluded and duplicates removed. The selected articles were assessed in terms of the method employed to measure light properties, reporting of light properties (source, wavelength, power, pulse frequency and beam area) and reporting of irradiation parameters (irradiance, exposure time, radiant energy and radiant exposure).

## Results

The initial search of the PubMed database resulted in 2295 and 177 articles according to the search terms employed (LLLT and photobiomodulation, respectively), which were filtered and screened (Fig. 1a, b) to 56 [31–86] and 18 [87–104] articles, respectively. Thus, a total of 74 articles formed the basis of this systematic review (Tables 1 and 2).

**Table 1:** The number of articles failing to report important LLLT/PBM parameters and information

Information not reported	References	Number articles for LLLT (total = 56)	Percentage (out of 56; %)	Percentage (out of 74 articles; %)	Number of articles for PBM (total = 18)	Percentage (out of 18; %)	Percentage (out of 74 articles; %)
LLLT articles							
Light source							
Light measurement method	[31–70]	40	71	73			
Wavelength	[48, 60]	2	3	3			
Source	[44, 54, 61]	3	5	8			
Power	[33, 34, 36, 40–42, 45, 48–50, 54, 58, 61–65, 67, 71–74]	22	39	41			
Pulse frequency	[33, 34, 36, 40, 42, 43, 45, 46, 48, 50, 51, 54–57, 59, 61–63, 67–70, 72–77]	30	54	52			
Beam area	[33, 34, 36, 40, 43, 45, 48–50, 54, 55, 58, 61–64, 67, 71–75, 77, 79]	24	43	41			
Irradiation parameters							
Irradiance	[31, 32, 38, 40, 43, 48, 51, 54, 55, 57, 58, 60, 62–64, 66, 68, 70, 76, 77, 80]	21	38	43			
Exposure time	[40, 50, 58, 61, 64, 70, 71, 74, 80]	9	16	16			
Energy (radiant)	[31, 33–36, 40–43, 45–50, 52–55, 58–65, 67–69, 71–75, 79, 81, 82]	40	71	74			
Radiant exposure	[38, 40, 49, 52, 54, 55, 63, 80]	8	14	16			
Articles with all information present	[83–86]	4	7	5			
Photobiomodulation articles							
Light source							
Light measurement method	[87–99]				14	77	73
Wavelength					0	0	3
Source	[95, 97, 100]				3	17	8
Power	[92, 94–98, 100, 101]				8	44	41
Pulse frequency	[88, 89, 93–98, 100–104]				13	72	52
Beam area	[92, 94, 95, 97, 98, 100]				6	33	41
Irradiation parameters							
Irradiance	[87, 89–91, 93, 95, 97, 99, 100, 102, 103]				11	61	43
Exposure time	[90, 93, 102]				3	16	16
Energy (radiant)	[87, 90–98, 100–104]				15	83	74
Radiant exposure	[89, 92, 98, 102]				4	22	16
Articles with all information present					0	0	5

**Table 2:** Reported irradiation parameters of recent (2014–2015) relevant literature in LLLT/photobiomodulation

Author/ref.	Method for light measurement	Light source	Dose	Conclusion
Low and level and light and therapy				
Wang et al. [34]	No info	Source, LED; wavelength, 660 nm; power, –; frequency, –; beam area, –	Irradiance, 3.5 mW/cm^2^; time, 6–24 min; energy, –; radiant exposure, 0–20 J/cm^2^	660 nm LED accelerated palatal wound healing, potentially via reducing reactive oxygen species production, facilitating angiogenesis and promoting provisional matrix and wound reorganisation.
Park et al. [36]	No info	Source, LED array; wavelength, 660 nm; power, –; frequency, –; beam area, –	Irradiance, 50 mW/cm^2^; time, 10 min/day; 20 days; energy, –; radiant exposure, 30 J/cm^2^	LLLT was a effective biostimulator of adipose derived stem cells in vascular regeneration
Moneib et al. [40]	No info	Source, erbium glass laser; wavelength, 1550 nm; power, –; frequency, –; beam area, –	Irradiance, –; time, –; energy, 30–40 mJ; radiant exposure, –	Treatment of active acne with 1550 nm erbium glass laser is effective.
Kansal et al. [43]	No info	Source, GaAs; wavelength, 904 nm; power, 12 mW; frequency, –; beam area, –	Irradiance, –; time, 10 s × 10 irradiations × 10 days; energy, –; radiant exposure, 4.2 J/cm^2^	No evidence of pathological changes in radiograph following LLLT.
Park et al. [45]	No info	Source, LED; wavelength, 660 nm; power, –; frequency, –; beam area, –	Irradiance, 50 mW/cm^2^; time, 10 min/day; 20 days; energy, –; radiant exposure, 30 J/cm^2^	LLLT is an effective biostimulator of spheroid human adipose derived mesenchymal stem cells in tissue regeneration.
Imaoka et al. [48]	No info	Source, SuperLizer; wavelength, –; power, –; frequency, –; beam area, –	Irradiance, –; time, 20 min; energy, radiant exposure, 7.64 J/cm^2^	The reduction of proinflammatory cytokine, IL-20 by LLLT suggests laser irradiation will be useful for rheumatoid arthritis therapy.
Lim et al. [49]	No info	Source, LED; wavelength, 635 nm; power, –; frequency, CW; beam area, –	Irradiance, 5 mW/cm^2^; time, 1 h; energy, –; radiant exposure, –	Direct and indirect exposure with 635 nm light can inhibit activation of pro-inflammatory mediators and may be clinically useful as an anti-inflammatory tool.
Asai et al. [50]	No info	Source, LED; wavelength, 630 nm; power, –; frequency, –; beam area, –	Irradiance, 20 mW/cm^2^; time, –; energy, –; radiant exposure, 0.5–3 J/cm^2^	Low-level red LED light can enhance MC3T3-E1 cell proliferation and osteogenic differentiation when the cells are cultured for a relatively long time.
Bavrina et al. [54]	No info	Source, –; wavelength, 356–530 nm; power, –; frequency, –; beam area, –	Irradiance, –; time, 15 min; energy, radiant exposure, –	Exposure to low-intensity incoherent red light after exposure to ionising radiation led to a decrease in the level of oxidative modification of protein and LPO products and diminished cellular manifestation of radiation sickness.
Ko et al. [55]	No info	Source, laser/LED; wavelength, 635 nm; power, 6 mW; frequency, –; beam area, –	Irradiance, –; time, 5 min (total); energy, –; radiant exposure, –	The use of low-level laser emitting toothbrush is a safe and effective treatment option for the management of dentine hypersensitivity.
Jun et al. [58]	No info	Source, Nd/YAG and Er/YAG; wavelength, 532 and 1064 nm; power, –; frequency, 2 and 10 Hz; spot size, 2 and 8 mm	Irradiance, –; time, –; energy, –; radiant exposure, 1.2–1.4 J/cm^2^	Combined therapy and Q-switched Nd/YAG alone were effective in reducing light solar lentigines.
Hiratsuka et al. [61]	No info	Source, –; wavelength, –; power, –; frequency, –; beam area, –	Irradiance, 63–333 μW/cm^2^; time, –; energy, –; radiant exposure, 2.76–36.13 J cm^−2^ day^−1^	Artificial light phototherapy suppressed dextran sulphate sodium-induced colitis in mice by suppression of pro-inflammatory cytokines and promotion of anti-inflammatory cytokines.
Felici et al. [62]	No info	Source, laser; wavelength, 1100–1800 nm; power, –; frequency, –; spot size, 10 × 15 or 10 × 30 mm	Irradiance, –; time, 4–10 s; energy, 34–50 J; radiant exposure, 5–65 J/cm^2^	The use of infrared radiation represents a valid alternative to surgical lifting but cannot replace it.
Gold et al. [63]	No info	Source, LED array; wavelength, 405–460 nm; power, –; frequency, –; beam area, –	Irradiance, –; time, 10 min/zone; energy, –; radiant exposure:	The Silk’n Blue device is a safe efficacious at home device for the treatment of mild to moderate inflammatory acne vulgaris.
Fioramonti et al. [64]	No info	Source, laser; wavelength, 500–1200 nm; power, –; frequency, 2.5 ms; beam area, –	Irradiance, –; time, –; energy, –; radiant exposure, 30–35 J/cm^2^	Intense pulsed light therapy is a safe and effective treatment for telangiectsias and striae rubra, also in the complex clinical condition of Behҁet’s disease.
Wang et al. [67]	No info	Source, LED; wavelength, 660 nm; power, –; frequency, –; spot area, –	Irradiance, 3.5 mW/cm^2^; time, 6–24 min; energy, –; radiant exposure, 5–20 J/cm^2^	LED light irradiation at 660 nm accelerated palatal wound healing, potentially via reducing reactive oxygen species production, facilitating angiogenesis and promoting provisional matrix and wound reorgainisation.
Wang et al. [69]	No info	Source, GaAlAs laser; wavelength, 808 nm; power, 170 mW; frequency, –; spot area, 3.8 cm^2^	Irradiance, 44.7 mW/cm^2^; time, 67.2–335.6 s; energy, –; radiant exposure, 3–15 J/cm^2^ × 20	Results suggest that 808 nm LLLT at a low energy density (3 J/cm^2^) and 8 J/cm^2^ is capable of enhancing sciatic nerve regeneration following a crush injury.
Pinheiro et al. [70]	No info	Source, diode laser and LED; wavelength, 780 nm (laser), 850 nm (LED); power, 70 mW (laser), 150 mW (LED); frequency, –; spot area, 0.4 cm^2^ (laser) and 0.5 cm^2^ (LED)	Irradiance, –; time, –; energy, –; radiant exposure, 20 J/cm^2^ (140 J/cm^2^ total)	The use of biphasic synthetic micro-granular HA + Beta − TCP graft improved the repair of bone defects, associated or not with laser or LED light.
Tsai et al. [71]	Power meter (Thorlabs, USA) to measure power densities	Source, GaAlAs; wavelength, 810 nm; power, –; frequency, CW; beam area, –	Irradiance, 20 mW/cm^2^; time, –; energy, –; radiant exposure, 1.5 J/cm^2^	LLLT moderately increased the uptake of mono-l-aspartyl chlorine(e6) and cellular ATP levels.
Gavish et al. [72]	LaserMate power-meter (coherent, Auburn group, Holland)	Source, diode laser; wavelength, 780 nm; power, –; frequency, –; beam area, –	Irradiance, 4 mW/cm^2^; time, 9mins; energy, –; radiant exposure, 2.16 J/cm^2^	LLLT prevented de novo development of abdominal aortic aneurysms (AAA) and also arrested further progression of pre-induced AAA and its associated deterioration in the biochemical integrity of the aortic wall.
Dungel et al. [73]	USB2000 spectrometer (Ocean Optics, FL)	Source, LED; wavelength, 470 nm and 629 nm; power, –; frequency, –; beam area, –	Irradiance, 50 mW/cm^2^; time, 10 min; energy, –; radiant exposure, 30 J/cm^2^	LED treatment of ischemia challenged tissue improved early wound healing by enhancing angiogenesis irrespective of wavelength.
Taflinski et al. [74]	Measured at Philips Research using a integrating sphere (Ulbricht)	Source, LED array; wavelength, 420 nm; power, –; frequency, –; beam area, –	Irradiance, 50 mW/cm^2^; time, –; energy, –; radiant exposure, 0–90 J/cm^2^	Observed effects are promising for a clinical use of blue light in the treatment or prevention of myofibroblast-mediated pathological condition such as tissue fibrosis and scleroderma or hypertrophic scarring.
Lanzafame et al. [77]	Ophir Nova Power meter (Model 30A-P-R-SH; Ophir Spiricon, UT)	Source, lasers and LEDs; wavelength, 655 nm; power, 5 mW; frequency, –; beam area, –	Irradiance, –; time, 25 min × 60; energy, 2.9 J/session; radiant exposure, 67 J/cm^2^	LLLT of the scalp at 655 nm significantly improved hair counts in women with androgenetic alopecia.
Tedford et al. [80]	Power Meter (OptoTest OP710-Si), thermal-based power detector with meter (ophir LD40-150, Ophir Nova), Optical power and wavelength detector (ILX lightwave OHM-6810B) and amplified photodiode (Thorlabs PDA10A)	Source, laser diode; wavelength, 660, 808 and 940 nm; power, 50 mW–70 W; frequency, PW and CW, –; beam diameter, 30 mm	Irradiance, –; time, –; energy, –; radiant exposure, –	The 808-nm wavelength light demonstrated superior CNS tissue penetration.
Photobiomodulation				
Freire et al. [85]	No info	Source, laser (GaInPAl; 660 nm) and LED (670 nm); wavelength, 660 and 670 nm; power, 40 mW (laser), 150 mW (LED); frequency, CW; beam area, 4 mm^2^ (laser) and 0.5 cm^2^ (LED)	Irradiance, –; time, 30 s (laser) and 16 s (LED); energy, 16 J; radiant exposure, 4.8 J/cm^2^ (laser) and 4 J/cm^2^ (LED)	The best results were obtained from the preventive laser and LED photobiomodulation groups; both groups were effective in diminishing the oral mucositis lesions.
Larkin-Kaiser et al. [89]	No info	Source, laser; wavelength, 800 and 970 nm; power, 3 W; frequency, –; beam area, –	Irradiance, –; time, 4 min; energy, 360 J; radiant exposure, –	Applied to skeletal muscle before resistance exercise, NIR light therapy effectively attenuated strength loss.
de Carvalho et al. [90]	No info	Source, GaAlAs (660 nm, laser) and InGaAlP (630 nm, LED); wavelength, 660 and 630 nm; power, 40 mW (laser) and 150 mW (LED); frequency, CW; beam area, 4 mm^2^ (laser) and 0.8 cm (LED) spot	Irradiance, –; time, –; energy, –; radiant exposure, 4.8 J/cm^2^	Laser and LED photobiomodulation were effective in accelerating the healing of formocresol-induced oral ulcers in both clinical and histological aspects.
Ban Frangez et al. [92]	No info	Source, LED; wavelength, 470, 625, 660 and 850 nm; power, –; frequency, kHz range; beam area, –	Irradiance, 2.16–8.23 mW/cm^2^; time, 3 min; energy, –; radiant exposure, –	Photobiomodulation using LED improved the sperm motility in asthenozoospermia regardless of wavelength.
Ramalho et al. [93]	No info	Source, InGaAlP laser; wavelength, 660 nm; power, 50 mW; frequency, –; spot area, 0.028 cm^2^	Irradiance, –; time, –; energy, –; radiant exposure, 3.57 J/cm^2^	Low- and high-power lasers can contribute positively to all steps of the indirect restorative treatment period.
Turrioni et al. [94]	No info	Source, LED (InGaN); wavelength, 450, 630 and 840 nm; power, –; frequency, –; beam area, –	Irradiance, 88 mW/cm^2^; time, 1 min 20 s and 8 min 40 s; energy, –; radiant exposure, 4 and 25 J/cm^2^	The infrared LED irradiation at an energy density of 4 J/cm^2^ and red LED at an energy density of 25 J/cm^2^ were the most effective parameters for transdentinal photobiomodulation of cultured ondontoblast-like cell.
Barbosa et al. [95]	No info	Source, –; wavelength, 670 nm; power, –; frequency, –; beam area, –	Irradiance, –; time, 3 min; energy, –; radiant exposure, 9 J/cm^2^	Morphological image analysis was used to quantify the extent of vaso-obliteration in oxygen-induced retinopathic vascular growth, the preventive effect (by photobiomodulation) of exposure during tissue development to near-infrared light (670 nm) and the lack of adverse effects due to exposure to NIR light.
Tang et al. [97]	No info	Source, –; wavelength, 670 nm; power, –; frequency, –; beam area, –	Irradiance, –; time, 160 s/day (2–9 months); energy, –; radiant exposure, 25 J/cm^2^	Photobiomodulation potentially offers a non-invasive and cost effective therapeutic option for patients with non-centre-involving diabetic macular oedema.
Ekizer et al. [98]	No info	Source, LED; wavelength, 618 nm; power, –; frequency, –; beam area, –	Irradiance, 20 mW/cm^2^; time, 20 min (200 min total); energy, –; radiant exposure, –	Photobiomodulation therapy has the potential of accelerating orthodontic tooth movement and inhibitory effects on orthodontically induced resorptive activity.
Di Marco et al. [100]	Calibrated sensor (Quantum devices, Barnfield, Wisconsin)	Source, –; wavelength, 670 nm; power, –; frequency, –; spot area, –	Irradiance, –; time, 3 min; energy, –; radiant exposure, 4–4.5 J/cm^2^	Detailed analysis suggests that there is a negative interaction between photobiomodulation and saffron when given simultaneously, with a consequent reduction of neuroprotection.
Pitzchke et al. [102]	Integrating sphere (LMS-200, 20-in. diameter, Labsphere, USA) and Power meter (detector 818P-010-12, driver 1918-R, Spectra Physics Newport)	Source, laser diode; wavelength, 671 and 808 nm; power, 0.8 W at 671 nm and 1 W at 808 nm; frequency, –; spot area, 1 cm^2^	Irradiance, –; time, –; energy, –; radiant exposure, –	Objective was to refine a possible treatment option for PD patients and validate the practicalities of light delivery and light dosimetry. The study demonstrates the possibility to illuminate deep brain tissues transcranially, transsphenoidally and via different application routes.

The majority (71/74; 96 %) of articles reported a positive effect following LLLT, with three articles [43, 70, 87] reporting nil effects following LLLT (Table 2) and none reporting negative effects. Of the 74 articles, 73 % (54/74) did not report methods for light measurement and relied on manufacturers’ information. Only 5 % (4/74) of articles reported a full set of data for the parameters/information assessed in this review [83–86]. For articles that did report light measurement methods, the most common was using a power meter (22 %; 16/74), or equivalent. Remarkably, only six (6/74; 8 %) employed a method that was able to measure spectral properties such as wavelength [39, 45, 54, 67, 68, 83]. Two articles (3 %; 2/74) failed to report even manufacturers quoted wavelength [48, 61]. Other parameters which were not reported were power (41 %; 30/74), beam area (41 %; 30/74), irradiance (43 %; 32/74), exposure time (16 %, 12/74), radiant energy (74 %; 55/74) and radiant exposure (fluence; 16 %; 12/74), and these are detailed in Tables 1 and 2.

## Discussion

The need for the measurement and standardisation of reported irradiation parameters has previously been emphasised [22, 29, 30], and it was proposed that eight key beam parameters should be reported in all LLLT studies [29]: wavelength, power, irradiation time, beam area (at the skin or culture surface), pulse parameters (frequency), anatomical location (skin colour, target location, i.e. depth below skin), number of treatments and the interval between treatments. Whilst other radiometric parameters such as divergence, depth of field, beam polarisation, coherence length, beam profile and spectral width are also important, the authors of that paper suggested those parameters were the minimum necessary for a repeatable scientific study [29]. Thus, in agreement with that paper, a ‘bare’ minimum approach should be adopted when describing beam parameters and a more thorough approach should utilise more technically demanding techniques such as beam profiling. The importance of describing light parameters and treatment protocol has also been emphasised in several other publications [22–28]. Thus, the focus of this current study was to provide an overview of the fundamental concepts of light measurement and set the basis for a proper evaluation of light properties. Therefore, this study has reviewed the properties directly related to light rather than treatment protocol (anatomical location, number of treatments, interval between treatments).

In the current literature search, 96 % of articles reported positive effects of LLLT with only three articles [43, 70, 87] showing no beneficial effect following LLLT. However, the number of articles that report measured information regarding light properties and irradiation parameters was remarkably low (27 %; Table 1) considering that the physics of light forms a fundamental basis of this therapeutic process. Even when light is measured, the methods employed are not always adequate to fully assess light properties; only 8 % of articles reported a method that was capable of characterising the spectral (wavelength) light output [73–75, 80, 82, 102]. The most common light measurement method used power or energy meters (Table 1), which is obviously an improvement on nil measurement; however, these devices are known to have significant limitations, which is discussed in ‘Photodiodes and power meters’.

Table 2 details the current state of light measurement and reporting of light parameters in LLLT studies where missing information does not allow for key parameters to be assessed. It is likely that these inadequacies are related to several factors that include expense of equipment, lack of expertise in equipment usage, poor appreciation of light properties and deficiencies of LLLT research standardisation. Consequently, this review continues by introducing fundamental concepts of light measurement and radiometric terms in attempt to explain their critical importance for LLLT research.

### Light: the basics

Over recent centuries, units of measurement have been established for quantifying and reporting the multitude of parameters that describe the wavelength, irradiance and incident beam area, distribution and energy of light. These parameters have significance for LLLT research, and when used properly will fully describe the ‘medicine’ (the light source and its properties) and the ‘dose’ (the irradiation parameters/protocol) and will improve reproducibility and information between researchers, manufacturers and clinicians.

### Radiometry

Radiometry is the measurement of electromagnetic radiation between approximately 10 and 1,000,000 nm. Within these wavelengths are the ultraviolet (UV; <400 nm), visible (~400–700 nm), near-infrared (~700–1400 nm) and infra-red (IR; >1400 nm) bands. Since LLLT experiments typically involve the application of light in the visible red and near-infrared region of the electromagnetic spectrum, radiometric terms should be employed to describe light properties and irradiation parameters that adequately depict key information needed for repeatable and reliable results between researchers, manufacturers and clinicians, which will ultimately improve clinical outcomes. Table 3 represents a summary of correct key terms, quantities and units that should be used in LLLT, although commonly incorrect terminology is stated. For example, studies will often report radiant exposure using the term energy density which actually describes a volumetric parameter rather than the amount of energy applied to a given area [66, 105–108] or use ambiguous terms such as intensity [108], which in radiometry can lead to confusion with ‘radiant intensity’ (the radiant power emitted, reflected, transmitted or received). Likewise, in LLLT, the term intensity does not distinguish whether the light is measured as ‘radiant exitance’ which is the amount of light leaving (emitted) from a surface, or ‘irradiance’ which is the amount of light arriving (irradiated) onto a surface; a subtle, yet critical consideration for accurate measurement of incident light at a specimen surface such as treated tissue or cell culture areas (‘Irradiance and radiant exitance’). Another example of a commonly used, largely ambiguous term is spot-size [39, 62, 91, 103] and the errors that may arise in assuming a circular beam area, which may not be representative of an elliptical laser speckle pattern (‘Beam area’). These ambiguous terms can potentially lead to misinterpretation of dosing parameters and poor reproducibility of data and should be avoided.

**Table 3:** Summary of quantities, symbols and units

Terminology commonly used	Correct terminology	Symbol	Equation of relation	Unit
Wavelength	Wavelength	*λ*		nm
Frequency	Pulse frequency	*ν*		Hz
Radiant energy	Radiant energy	*Q*		Joule, J
Energy density	*Not applicable*	*u*		J/cm^3^
Energy density/fluence	Radiant exposure /(radiant) fluence	*H*	*∫ E* d*t*	J/cm^2^
Power (flux)	Power/radiant flux)	*Φ*	d*Q/*d*t*	watt, W
Spot size	Beam area	*A*		cm^2^
Power density/intensity	Irradiance	*E*	d*φ/*d*A*	W/cm^2^
Intensity	Radiant exitance	*M*		W/cm2
Exposure time/duration	Exposure time/duration	*t*		s

### Spectral quantities

Radiometric quantities often have a spectral (or wavelength) variable. The spectral variable describes the distribution of these quantities with respect to their representative wavelengths: the total irradiance of a light source is defined by the irradiance at each individual wavelength. Spectral measurements are particularly important for chemical or biological applications, as the knowledge of spectral content is often vital in choosing or interpreting the effects of a particular light source. This is potentially critical as popular work by Karu [109] suggests light of appropriate wavelength is absorbed by copper complexes within the mitochondrial enzyme, cytochrome *c* oxidase (CCO), which then causes the release of bound nitric oxide leading to further downstream cell signalling effects [110, 111]. Therefore, there must be an effective spectral overlap between the absorption of CCO and laser/LED emission for therapeutic LLLT. Consequently, not only is it important to characterise the spectral properties of the light source but also the absorbance profile of materials or tissue that can potentially absorb the therapeutic window of emitted light. It follows that accurate measurement and reporting of spectral information (peak wavelength, spectral irradiance, spectral half-width and absorption profiles) would confirm (or otherwise) the conclusions made in LLLT studies.

### Light, quantities, units and symbols

#### Radiant energy

Electromagnetic radiation can be considered as both a wave and a particle (depending on how it is measured), which transports energy through space. This energy can be absorbed by physical objects and converted into other forms such as thermal or electrical energy (solar cells). For example, in photographic light meters, incident visible light causes electric current flow when the radiant light energy is transferred to electrons as kinetic energy, from which light power can be inferred (‘Photodiodes and power meters’). Similarly, for LLLT, light is transferred to cells as radiant energy that modulates cellular responses via CCO absorption and is analogous to photosynthesis, whereby the action of light, and light alone, directly stimulates cell responses. Whilst radiant energy is important [112], the parameter alone is not enough to determine treatment efficacy since an infinite combination of irradiance and exposure could lead to similar radiant energies. Radiant energy is denoted as *Q* and expressed in *Joules* (J)*.* Spectral radiant energy accounts for monochromatic (such as a single wavelength laser) and polychromatic sources (for example, lights which emit over a range of wavelengths) and is defined as radiant energy per unit wavelength interval at wavelength, *λ*:

1 $$ {Q}_{\lambda } = \frac{dQ}{d\lambda } $$

The units of spectral radiant energy are *Joules per nanometre* (J/nm)

#### Radiant flux or radiant power

**Fig. 2:**
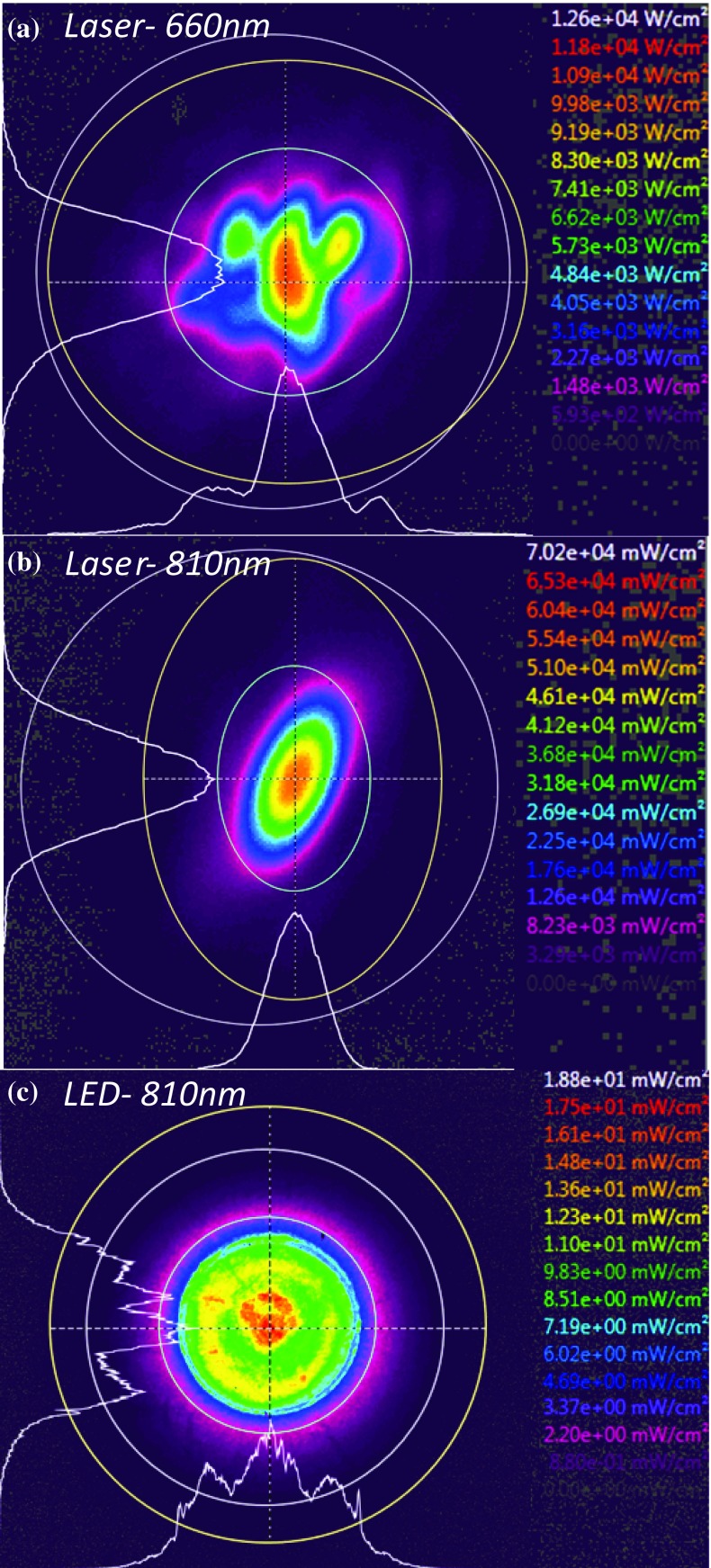
Examples of spatial distribution of irradiance in lasers and LED lights where the highest to lowest irradiance is represented by the *rainbow colours*, *red* to *violet*, respectively, for **a** 660 nm laser, **b** 810 nm laser and **c** 810 nm LED

‘Flux’ or ‘power’ describes the time rate of flow of radiant energy. This is a parameter that is usually reported within LLLT literature through manufacturers’ information or measured using power/energy meters (Tables 1 and 2). Metaphorically, this describes the ‘potency’ of the light and although important, does not provide adequate information concerning spectral and spatial distribution of the energy or the actual irradiance delivered to the target site. Lack of spatial information assumes uniform irradiance over the output area [113, 114], which can be far from accurate, especially considering the true irradiance across an active beam area (Fig. 2). This may differ according to the type of light source, e.g. the common elliptical profile of lasers (Fig. 2b) or the non-unform irradiance distribution of LEDs (Fig. 2c) and the distance from the light tip to the target area. Although power or radiant flux should be reported, it only partially describes irradiation parameters that are necessary for complete information relevant to LLLT research. Radiant power or flux has units of *Joules per second* (J/s) *or watts* (W) and is defined as:

2 $$ \phi = \frac{dQ}{dt} $$

The radiant flux per unit wavelength interval at wavelength, *λ* is given the term spectral radiant flux and is defined as:

3 $$ {\phi}_{\lambda } = \frac{d\phi }{d\lambda } $$

and measured in *watts per nanometre* (W/nm).

#### Beam area

Beam area is often referred to as ‘spot-size’ in the LLLT literature [39, 62, 91, 103]. However, the term beam area should be preferred over spot-size and reported in *square centimetres.* The term spot is usually descriptive of a circular shape and size is ambiguous although units may remove ambiguity. As mentioned previously, lasers may emit an elliptical beam, which would significantly affect the area calculation (Fig. 2) and lead to misinterpretation of irradiation parameters. In cases where the beam area is non-circular, or of circular Gaussian, the beam area and/or diameter can be accurately determined using techniques such as beam profilometry which will be discussed in ‘Light measurement/detectors’. Nonetheless, this review finds that a significant number of LLLT studies fail to report beam area (Tables 1 and 2), a key parameter that should be reported in all LLLT studies.

The radiant beam area acting on a target site is likely to significantly influence biological response in both *in vivo, in vitro* and clinical studies. Although systemic and local responses to LLLT irradiation have been reported [115] *in vivo*, beam area is also important for dosing and radiometric calculations. *In vitro*, a localised effect of light irradiation is likely to result in a significant biological response. Consequently, if the beam area is much smaller than the target culture area, then only a proportion of the host cells will be irradiated, attenuating the measured biological response and possibly resulting in a false-negative result. Therefore, a suggested good practice would be to ensure the whole culture well is irradiated evenly with a round, flat top beam.

#### Irradiance and radiant exitance

**Fig. 3:**
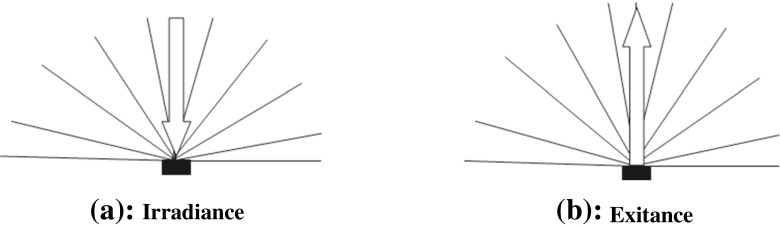
The definitions of radiant flux density arriving (**a** irradiance) or leaving (**b** exitance) a surface (the *lines* represent rays of light travelling in the direction of the *arrow*)

The radiant flux per unit area received by a surface from any direction can be termed irradiance (Fig. 3a). However, sometimes this is confusingly termed power density or intensity in the LLLT literature, and this does not distinguish between irradiance or light arriving (irradiance) or that leaving a surface (exitance). Irradiance is defined as:

4 $$ E = \frac{d\phi }{dA} $$

where *dϕ* is the radiant flux and *dA* is differential area. The measured flux can also be that leaving the surface from any direction due to emission and/or reflection (Fig. 3b) and is given the term radiant exitance and defined as:

5 $$ M = \frac{d\phi }{dA} $$

where *dϕ* is the radiant flux leaving and *dA* is differential area where *dϕ* is leaving from. A possible use of this terminology in LLLT could be to describe reflection off biological tissue such as dentine, bone and other tissues.

Irradiance can be measured in space and is usually measured in *watts per square centimetre*. This includes the surfaces of physical objects, i.e. tissue or cell culture samples, and the space occupied between them, i.e. air or through tissue thickness. This is particularly important when characterising lights that are highly divergent, as found in typical LED sources, or through tissue which is highly scattering.

Commonly, irradiance is not verified by researchers and only reported from manufacturers’ quoted values or is calculated using values gained from inadequate measurement tools (Tables 1 and 2). Manufacturers’ values are typically measured at the aperture or fibre tip, but because the beams are highly divergent, they are not useful for studies where the beam may be projected to a target such as in an in vitro study. Manufacturers’ specific methods of measurement are also rarely divulged. Irradiation, *in vivo* or in *in vitro* cultures, likely occurs through tissue or culture plastics at distances greater than 0 mm. Therefore, replicating and measuring key light parameters at, or through, relevant targets at specific distances and geometry is far more accurate and clinically and experimentally relevant than using manufacturers’ data alone.

Spectral irradiance is the radiant flux per unit wavelength interval at wavelength *λ* and can be defined for both irradiance and radiant exitance with the following equations:

6 $$ {E}_{\lambda } = \frac{dE}{d\lambda } $$

7 $$ {M}_{\lambda } = \frac{dM}{d\lambda } $$

Spectral irradiance is measured in *watts per square centimetre per nanometre*.

#### Exposure duration

Although the most straightforward irradiation parameter to measure, exposure time (*s*) is not always reported in the literature. Sixteen per cent (12/74) of articles reviewed in this study failed to report exposure duration (Tables 1 and 2), which is likely due to a common misconception that wavelength, flux (radiant energy) and fluence are all that are necessary to replicate a successful treatment [29]. Exposure duration is a key component of ‘dose’, which is the product of irradiance and exposure time and should always be separately defined (Section ‘Radiant exposure and exposure reciprocity’).

In addition to reporting exposure time, when multiple exposures are performed, the number of treatment sites, the number of exposures and the interval between exposures should also be reported in order to fully describe the treatment protocol [29, 30].

#### Radiant exposure and exposure reciprocity

The energy delivered per unit area of cells during light stimulation for LLLT is also an important parameter since the efficacy of the treatment would depend on the irradiance delivered over a given area. The quantity areal, measured in *joules per square centimetre* and is often incorrectly termed energy density, should only be used for volumetric energy deposition (J cm^−3^). The proper terminology for the total amount of energy delivered per unit area is ‘radiant exposure’, or more commonly termed fluence in the LLLT literature, where *H* (the radiant exposure or fluence) is defined as the integral of the irradiance from Eq. 4

8 $$ H={\displaystyle {\int}_0^TE\ dt} $$

However, many researchers merely quote radiant exposure (sic. energy density) as an expression of dose within the literature with missing irradiance (W/cm^2^) or exposure duration (s) values (or even both; Tables 1 and 2). This is potentially unreliable, as it assumes an inverse correlation between the effects of irradiance and exposure duration.

The Bunsen-Roscoe ‘Law of Reciprocity’ states that photochemical reactions will be independent of irradiance and exposure time with the effects being directly proportional to the total energy delivered [116–118]. Although it can be assumed that this law is valid for photochemical reactions within a certain dose range, photobiological responses of cells and tissue usually involve a sequence of interacting biological reactions making a linear dose-time relationship less likely. A true reciprocal relationship between irradiance and time would achieve similar therapeutic effects regardless of how radiant exposure was achieved (e.g. 20 s at 100 mW/cm^2^ would exhibit similar therapeutic effects compared with 200 s at 10 mW/cm^2^ or 80 s at 25 mW/cm^2^). However, although an effective radiant exposure for a specific cell type is an important, and a largely unknown quantity in LLLT, the individual parameters (irradiance and time) are critical and should also be defined. Notably, if the irradiance is too low and/or the delivery time too short, any significant beneficial effect may not be realised or even reduced [19, 81]. Furthermore, if the irradiance is too high or the irradiation time is too long, any significant benefit may also be attributed to heat, or even sometimes produce inhibitory, rather than therapeutic effects [19, 68]. Thus, any useful concept of exposure reciprocity may not be applicable in biological systems such as LLLT, since treatment modalities may only be effective within a window of specific irradiation parameters [19]. However, using similar radiant exposure by varying the combination of irradiance and time, and its effect on stimulatory/inhibitory cell responses is not fully understood and warrants a systematic approach to further understanding of the photobiomodulation of different cell types.

#### Pulse frequency

The pulse frequency is the number of pulses of a repeating signal in a specific time frame and is usually measured pulse per second (Hz), thus pulse operation of lasers or LEDs is not classified as a continuous wave. This type of operation is beneficial for heat dissipation and to achieve high peak irradiances, but since there is an on/off period, dosing parameters such as radiant energy and radiant exposure are affected which may affect the efficacy of LLLT [52, 64, 68]. For example, if the irradiation was pulsed to deliver light at 0.5 s intervals, then only half the energy would be delivered compared with continuous delivery at similar irradiance and exposure time. Thus, when pulsing regimes are utilised, the peak irradiance should be defined along with pulse frequency and the on/off durations as previously recommended [29, 30].

### Light measurement/detectors

#### Spectro(radio)meters

**Fig. 4:**
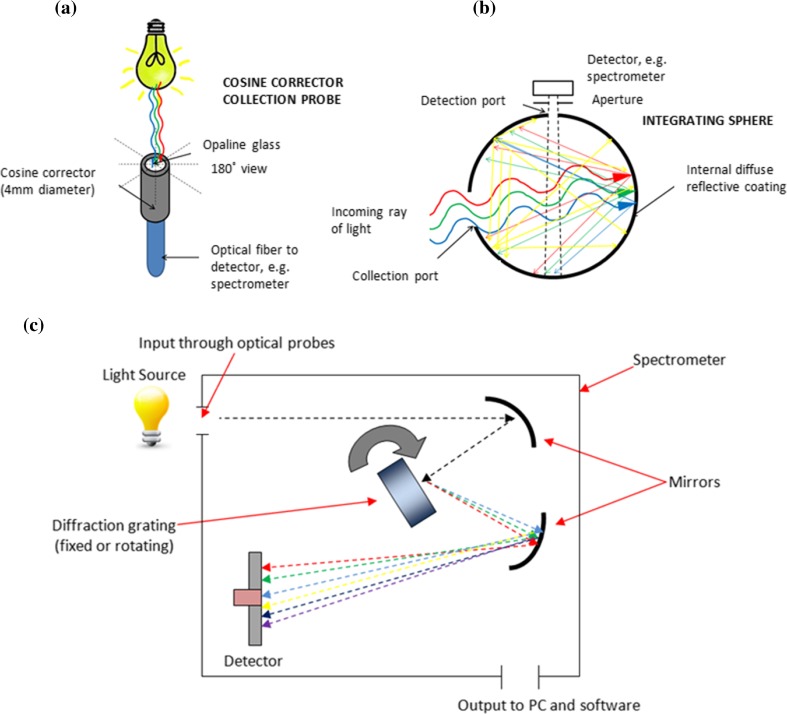
Schematic representation of the internal workings of an **a** integrating sphere showing the 360° collection of light; **b** a cosine corrector probe allowing a 180° field of view; and **c** the internal workings of a UV–vis spectrometer

A spectrometer is an instrument used to measure the properties of light over specific portions of the electromagnetic spectrum and provides a useful system to analyse spectral characteristics critical for LLLT research. Spectrometers are coupled with flexible, transparent optical fibres of varying diameters made from high-quality glass that function as waveguides or light guides to transmit light between the two fibre ends. Opaline cosine correctors are usually attached, which have diffusing material apertures allowing light measurement normal to its surface with 180° field of view (Fig. 4a). Whilst cosine corrector probes provide a cheap, versatile, robust and reliable method of light measurement, the measurement accuracy is limited when analysing large light sources due to its small collection area and its 180° field of view. Alternatively, integrating spheres of varying diameter and port size (dependent upon the source size) can be used, which consist of hollow spherical cavities covered with diffuse white reflective coating. Spheres can be used to capture and measure light radiated in all directions from the light source as light scattered by the interior of the integrating sphere is evenly distributed over all angles (Fig. 4b). However, measurements using integrating spheres are limited by the size of the sphere and the size of the light source intended to be measured. Nevertheless, the fibres and cosine corrector (or integrating sphere) collectively become an optical probe, which can be calibrated using a photometric standard or calibrated light source to National Institute of Standards and Technology (NIST) standards providing an accurate measurement system known as a spectroradiometer.

Light is captured through the cosine corrector or integrating sphere and travels through the optical fibre into the spectrometer. The core of the spectrometer is formed by a diffraction grating which splits radiant light into its spectral components and projects the diffracted elements onto a detector. Computer software is used to calculate all radiometric, photometric and colourimetric quantities from spectral data. Two types of spectrometers exist, an array type, which has a fixed diffraction grating and a detector array, and a scanning spectrometer, which has a single detector and a rotating diffraction grating (Fig. 4c). Spectrometers are popular light measurement systems for many aspects of photonics research although rarely used in LLLT studies (Table 2). However, there are other limitations of fibre-coupled spectrometers which use cosine correctors and integrating spheres, primarily that power uniformity within the incident beam is assumed and the power distribution of light across the exit diameter of large light sources cannot be measured [114]. For example, using a typical cosine corrector diameter of 4 mm to measure an incident beam diameter of 10 mm, the outer 6 mm of the beam will not be captured by the sensor. Light sources used for LLLT typically have a Gaussian distribution and therefore if the irradiance is measured centrally, power is assumed to be equal over the whole area and the measured irradiance would be overestimated. Error is increased with the increasing ratio of beam diameter to probe diameter (or vice versa) and researchers need to cautiously interpret data in such situations. Ideally, researchers should employ methods that will adequately measure all of the light by considering the projected beam area on a target and the distance that the beam is applied from. For example, if LLLT studies are performed by irradiating culture dishes or tissue samples from a specific distance, then the experimental light measurement methods should simulate this to accurately analyse light properties at the target site (irradiance), i.e. measure light received by cells, not what the light outputs. The effects of absorption, scattering and reflection by media, cell culture plasticware and other materials/tissue on spectral irradiance at the target site are critical and should be carefully considered, i.e. for cell culture work, the irradiance delivered on the culture area through plasticware should be measured to accurately determine the irradiance delivered to cells.

Spectrometers can also be used to measure absorption characteristics of specific cellular chromophores or photorecepetors localised in the mitochondria that are responsible for the absorption of light. A light source emitting multiple wavelengths is focused on to a sample which attenuates light through absorption, scattering and reflection of the incident light. The action spectra, a plot of relative effectiveness of different wavelengths, which is believed to mimic the absorption spectrum of CCO, has been reported by Karu et al. [119] and indicates several effective bands relating to the copper complexes of CCO. Thus, by recording this attenuation of light for various wavelengths, an absorption spectrum can be obtained and potential therapeutic windows for LLLT can be identified for specific tissue.

#### Photodiodes and power meters

A photodiode (detector) is responsive to optical input from UV to near infrared radiation and operates as a photoelectric converter generating a current that is proportional to the incident light. A photon of sufficient energy creates an electron hole pair by a mechanism known as the inner photoelectric effect that is dependent upon the efficiency of the photodiode. Quantum efficiency is dependent upon many factors, but in general if the energy of the photon is greater than the energy gap of the device, these photons will be absorbed very near the surface where the recombination rate is high and will contribute to a photocurrent. Thus, the photocurrent produced by the photodiode is proportional to the power of the light which can be measured directly by a ‘power meter’ which uses an operational amplifier circuit known as a transimpedance amplifier.

Although this type of measurement system is most popular within LLLT literature (Table 1), measurements from these devices should be interpreted cautiously. The spectral sensitivity differs with wavelength due to the quantum efficiency of the photodiode and generally has a better response at longer wavelengths. Thus, if broadband light sources are measured, the power emitted at short and longer wavelengths maybe be under- or over-estimated, respectively. Photodiodes also assume power uniformity across the beam and do not therefore effectively characterise the distribution of light for the same reasons described previously (‘Spectro(radio)meters’). Although reproducible measurements can be made at the sub-picoampere regime, the response time is limited by the sensor size, which slows as surface area increases. Furthermore, the detectors are usually made from fragile or sensitive materials such as silicon (Si; 190–1100 nm), germanium (Ge; 400–1700 nm), indium gallium arsenide (InGa; 800–2600 nm), lead(II) sulphide (<1000–3500 nm) or mercury cadmium telluride (400–14000 nm) which can be prone to damage and therefore measurements usually must be made without contact with the detector. Consequently, even small distances are likely to result in a loss of power due to divergence which will reduce the measured power. More so and unlike spectrometers, photodiodes do not provide spectral information and are usually only limited to power readings (W) and crude irradiance measurements (W/cm^2^) based on the sensor size or inputted tip or beam area values. However, photodiodes are relatively insensitive to temperature fluctuation (not critical for LLLT as low powered sources are used) and their main and unique advantage lies in their ability to measure very small optical powers which is specifically useful for basic light characterisation in LLLT studies.

#### Thermopiles

Thermopiles are essentially thermal sensors, which are best suited for measuring constant wave (CW) laser power, average power in pulsed lasers or the energy of long pulses. Thermopiles are robust, reliable and are a well-established method to measure light energy. They can be considered as an array of miniature thermocouple junctions connected in series as differential pairs. These differential pairs make up cold and hot junctions that are connected by alternating n-type and p-type materials. Thermopiles operate by using temperature differences to create a voltage, which is correlated to the temperature gradient between the hot and cold junctions and proportional to the light energy. These systems are particularly useful for measuring high powered sources (>1 Watt) which can damage other types of sensors. Thermopiles are made from materials such as antimony (Sb), bismuth (Bi), poly-silicon gold (Au) or alumininium (Al) and operate over a broad spectral range (200–20000 nm). Thermopiles tend to be more accurate than photodiodes but measurement sensitivity is reduced at low power. Since thermopiles work by using temperature gradients, they can be used to map irradiance distribution and offer uniform spatial response that is unaffected by changes in beam size, position or uniformity unlike photodiodes and spectrometer based systems which rely on inputted beam area values for irradiance calculation. However, although this type of sensor validly characterises the distribution of light and provides an accurate measure of important light properties such as irradiance and beam area, like the photodiode, it does not provide spectral information. Furthermore, response times are slow (generally a few seconds), which could be problematic in time-dependent experiments and thus are only really capable of measuring average powers. In addition, since the measurement is based on heat exchange, rapid fluctuations in housing temperature will decrease accuracy.

#### Charge coupled device cameras and beam profilometry

**Fig. 5:**
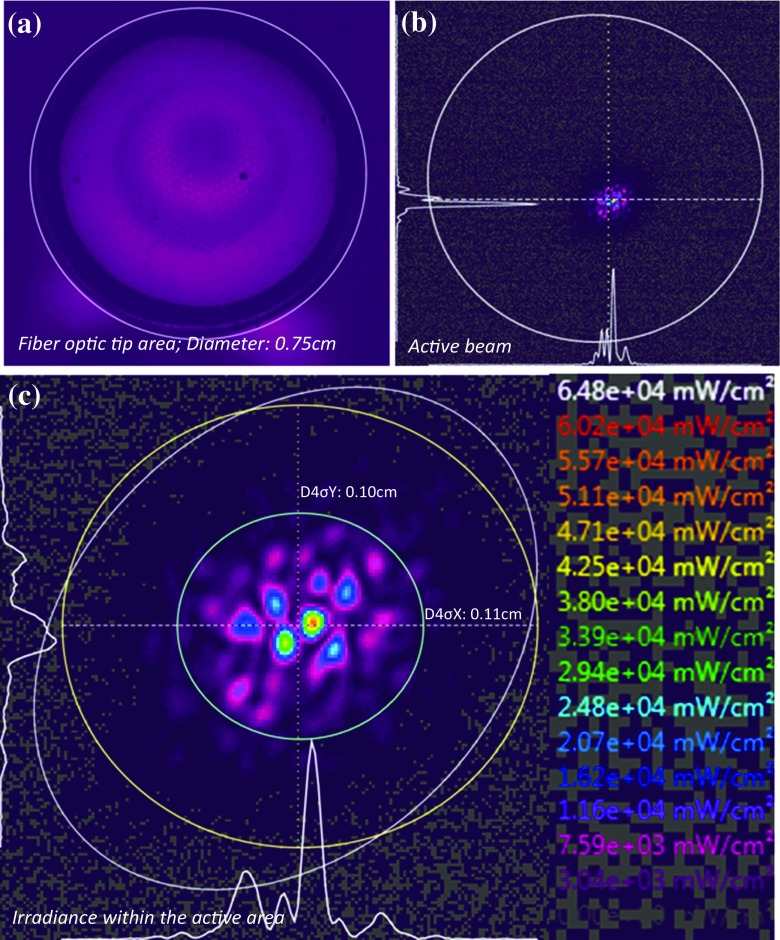
A 2D beam profile image of a LLLT laser device: **a** an image of the actual tip area used for light delivery, **b** the actual active beam area and the location of the beam within the fibre optic tip and **c** the laser ‘speckle’ beam pattern of the devices and its active beam diameter/area

A charge coupled device (CCD) is an integrated circuit etched onto a silicon surface forming light sensitive elements called pixels. Photons incident on this surface generate charge that is converted into a digital copy of the light pattern. Following appropriate calibration, beam profilometry is very useful for characterisation and quantification of power distribution and irradiance of a given light source and has the advantages of both photodiodes (good response time and unaffected by temperature) and thermopiles (unaffected by beam diameter, good sensitivity and spatial distribution of power and irradiance, which can be used over a large range of power outputs). Light can be collected through lenses and directed onto the CCD sensor which then creates a digital image of the beam. Calibration using pre-determined power values may then be used to calculate the average power delivered to each pixel within the defined beam area to create a mapped irradiance image [120]. This is known as the top-hat factor and can be used to characterise the degree of spatial [114, 120–122] and spectral [123] uniformity of the power distribution. For system calibration, if the total measured power is calculated (using, for example, a photodiode or thermopile), the power received by each pixel in the detector’s diode array can be calibrated to generate a 2- or 3-dimensional map of irradiance distribution across the active beam area. Therefore, the beam area can be accurately calculated rather than only measuring the light delivery tip diameter by crude methods such as calipers. For example, if the tip size of a LLLT laser device is much larger (7.5 mm diameter) than the actual beam diameter (~0.11 mm diameter; Fig. 5), erroneous irradiance values are inevitably obtained if the active beam area is assumed to be the same as the tip diameter. Thus, the need for standardised beam area calculation is required in LLLT, preferably using the ISO standard method (D4σ or second moment width; ISO 11145 3.5.2 [122]) or 1/e^2^ as suggested previously [29, 30].

Although CCD cameras and beam profilers are widely used for a variety of applications including dental research [114], this method has only been utilised in a limited number of LLLT studies [82, 120]. However, whilst beam profilers provide a relatively accurate measurement system, they are also unable to readily provide spectral information and are sensitive to spectral variation. Accurate and reliable test centres for LLLT research would have a suite of complimentary equipment including spectrometers or integrating spheres, photodiodes and beam profilers.

## Safety requirements for LLLT

Whilst the perceivable dangers of LLLT are mainly related to retinal damage (both clinician and patient) and skin burn (mainly related to shorter UV wavelengths), the safety of LLLT is well documented in a number of standards such as US Code of Federal Regulations, American National Standards Institute and the International Standards Manual, and other laser safety books and review articles [21, 22]. This includes ‘The Guidelines for Skin Exposure to Light’ in the International Standards Manual (IEC-825) which states that an exposure of less than 200 mW/cm^2^ is safe, and the marketing and the use of therapeutic LLLT is approved by the Food and Drug Administration. Preventative measures such as safety googles should always be utilised to minimise any risks and therapeutic devices may utilise high-powered light sources (>500 mW) may be spread over larger areas to fall within the recommended irradiance exposure limits. The operation of high-powered light sources may also be compensated by pulsing which may reduce the risk of any adverse effects caused by heating as discussed previously.

## Recommendations

LLLT has generated markedly increasing interest in a wide variety of biomedical disciplines. However, researchers frequently report LLLT studies that have inadequate information regarding light properties and use ambiguous terminology. Thus, it is increasingly difficult to compare and contrast study outcomes, which hinders the progress in this field. Researchers working in LLLT should utilise a minimal set of standard criteria for light measurement and reporting of radiometric data that are necessary for a repeatable scientific study and are sufficient to compare and contrast study outcomes. These include ten key parameter: wavelength, power, irradiation time, beam area (at the skin or culture surface), radiant energy, radiant exposure, pulse parameters, number of treatments, interval between treatments and anatomical location, which will improve the information available to other researchers. A similar approach is utilised in other biomedical research areas such as mesenchymal stromal stem cell (MSC) research which has a set of standard criterion to foster a more uniform characterisation of MSC and facilitate better exchange of data among investigators [124].

The measurement of light is fundamentally important for LLLT research, thus researchers should not merely rely on manufacturers information which is what is reportedly routinely practiced in many LLLT studies (Tables 1 and 2). Instead, researchers should use a combination of complimentary methods that will accurately describe the ten key parameters previously mentioned. For example, to describe the spectral output of a light source, spectrometer-based systems should be used, improving accuracy by employing integrating spheres to capture all of the light rather than cosine correctors. Similarly, to describe spatial distribution of power and irradiance, beam profilers or thermopiles could be employed. Further, as good practice, light property information should be fully reported in a standardised form as recommended previously [22, 29]. The terminology should also be consistent from study to study, which will make comparison and experimental repetition more straightforward. For example, instead of power density, the term irradiance should be used; instead of energy density, use radiant exposure or fluence, and so on (Table 3). Finally, the units should also be appropriately assigned, e.g. watts per square centimetre or milliwatts per square centimetre for irradiance depending on the output of the light source. Although these recommendations will probably require modification as new knowledge, technology and techniques unfold, they provide a minimal standard criteria that will facilitate a better exchange of information within LLLT which could ‘drive’ this field forward.

## Conclusions

It is apparent that a relatively poor appreciation of radiometric properties exists within the literature associated with LLLT. Proper radiometric measurements are fundamental for this area of research and although it may appear straightforward, concepts and appropriate measurement techniques are commonly misunderstood, or ignored. Furthermore, the literature suffers greatly from missing information such as wavelength, power, pulse parameters, beam area, beam profile information, irradiance, exposure time, radiant exposure and evidence of calibrated measurement tools, making reliability questionable and reproducibility difficult all of which weakens the strength of conclusions potentially giving rise to false or nil results. The persistence of misunderstanding, inadequate experimentation and inaccurate reporting of radiometric data within LLLT literature has, and will continue to affect the reliability of LLLT information shared between scientists, manufacturers and clinicians. Ultimately, accurate measurements and reporting of light properties is essential to fully understand the potential beneficial biological mechanisms of LLLT, which could be achieved by following the recommendations of this review.
